# Exposure assessment for the abandoned metal mine area contaminated by arsenic

**DOI:** 10.1007/s10653-019-00296-5

**Published:** 2019-04-23

**Authors:** Jun Young Chang, Seung Chul Ahn, Jung Sub Lee, Jee-Young Kim, A-Ra Jung, Jaeseon Park, Jong-Woo Choi, Seung Do Yu

**Affiliations:** 1 Environmental Health Research Division, National Institute of Environmental Research, 42, Hwangyeong-ro, Seo-gu, Incheon, Republic of Korea; 2 Indoor Environment and Noise Research Division, National Institute of Environmental Research, Incheon, Republic of Korea; 3 Wonju Regional Environmental Office, Wonju, Republic of Korea; 4 Environmental Measurement and Analysis Center, National Institute of Environmental Research, Incheon, Republic of Korea

**Keywords:** Arsenic, Exposure assessment, Abandoned metal mine, Stable Pb isotope ratio

## Abstract

Among the results of community health impact assessments completed in 2014, residents of the Indae abandoned metal mine area showed high average urinary concentrations of harmful arsenic (As), at 148.9 µg/L. The concentration of harmful As was derived as the sum of As(V), As(III), MMA, and DMA concentrations known to be toxic. In this area, mining hazard prevention work was not carried out and the pollution source was neglected, and the health effect of the residents due to arsenic exposure was concerned. We re-assessed As exposure levels and tried to identify exposure factors for residents of this area. Analysis of the soil, sediment, and river water to assess the association between the soil of the Indae abandoned metal mine area and the soil in residential areas confirmed a correlation between Pb and As concentrations in the soil. Since Pb and As behave similarly, the use of the stable Pb isotope ratio for assessment of the pollution source tracking was validated. In the 3-isotope plot (^207/206^Pb vs. ^208/206^Pb) of soil samples in this area, a stable Pb isotope ratio was located on the same trend line, which confirmed that the soil in the residential area was within the area of influence of the Indae abandoned metal mine. Therefore, we judged that the pollution source of As was the Indae abandoned metal mine. The results by As species were As (III) 1.45 μg/L, As (V) 0.74 μg/L, monomethylarsonic acid (MMA) 2.43 μg/L, dimethylarsinic acid (DMA) 27.63 μg/L, and arsenobetaine 88.62 μg/L. The urinary harmful As was 31.92 μg/L, much lower than the 148.9 μg/L reported in a 2014 survey, due to the implementation of a multi-regional water supply in November 2014 that restricted As exposure through drinking river water. However, concerns remain over chronic exposure to As because As in river water used for farming and in agricultural soil still exceeds environmental standards; thus, ongoing work to address hazards from former mining areas and continued environmental monitoring is necessary.

## Introduction

Arsenic (As), a naturally occurring element distributed widely in the Earth’s crust, is a metalloid with similar properties to those of metals. Although As has been used extensively as an ingredient in wood preservatives, pesticides, automotive storage batteries, semiconductors, and light-emitting diodes, limitations are being placed on its use due to concerns over its toxicity (ATSDR 2007; WHO 2001).

As is a carcinogen that can cause lung and skin cancer, and is a substance with strong toxicity that can cause cardiovascular, skin, respiratory, and neurological diseases (ATSDR 2007; WHO 2001).The International Agency for Research on Cancer (IARC) of the World Health Organization (WHO) classified As and As compounds as Group 1 carcinogens (carcinogenic to humans) in 1987, As in drinking water as a Group 1 carcinogen in 2004, and monomethylarsonic acid (MMA) and dimethylarsinic acid (DMA) as Group 2B carcinogens (possibly carcinogenic to humans) in 2012 (WHO IARC 2004, 2012; Hsu et al. 2011).

The toxicity of As varies according to type, whether inorganic or organic. Inorganic As includes arsenous acid (As(III)) and arsenic acid (As(V)), while organic As includes MMA, DMA, arsenobetaine (AsB), and arsenocholine (AsC). Generally, inorganic As has a stronger toxicity than that of organic As (Tseng. 2007). However, methylated trivalent species, MMA^III^ and DMA^III^ produced as intermediate in the metabolic processing of inorganic As may be responsible for carcinogenic effects (Yamanaka et al. 2004). As(III) has a particularly strong toxicity since it has better reactivity with cells than As(V) (Naranmandura et al. 2006) (Liu et al. 2013). AsB and AsC are generally considered to be non-toxic. Therefore, the toxicity and carcinogenicity of As depend not only on the total concentration but also on the different As species (Pizarro et al. 2003) (Liu et al. 2013).

As in the environment usually exists in an inorganic form. Inorganic As is absorbed into the body by various routes, such as inhalation, oral ingestion, and skin contact, where it is metabolized by a methylation process and converted to organic As in the forms of MMA and DMA, much of which is excreted through the urine within 3 days. This metabolic process is a pathway through which the toxicity of As is reduced (ATSDR 2007). In contrast, organic As, such as AsB, is excreted through the urine without undergoing any such metabolic process (ATSDR 2007).

As illustrated in cases from China (Wei et al. 2018; Soldatova et al. 2018), India (Das et al. 1996), Pakistan (Fatmi et al. 2013) and Taiwan (Lan et al. 2011), residents who use As-contaminated groundwater as drinking water may suffer fatal health consequences due to As exposure.

Most of the metal mines in Korea were developed prior to 1940; however, since the 1980s, they have been closed due to a decline in their economic feasibility and some of these abandoned mines contain mine waste (KMOE 2017a). To protect the health of Korean citizens, the Korean Ministry of the Environment (KMOE) began conducting soil contamination surveys on abandoned metal mines throughout Korea from 1992, and mining hazard prevention programs are being implemented for abandoned metal mines with confirmed contamination through relevant departments, such as the Korean Ministry of Trade, Industry, and Energy (KMOE 2017a). Up to 2016, surveys of 1536 abandoned metal mines had been completed, and among them, 672 abandoned metal mines exceeded the worrisome level of soil contamination in Korea for arsenic (As) (KMOE, 25 mg/kg) and cadmium (Cd) (KMOE, 4 mg/kg) (KMOE 2016), while heavy metals have also been detected in river water, sediment, and agricultural products (KMOE 2017a). Mining hazard prevention work has been completed for 192 of these abandoned metal mines, and there are plans to continue to this work in the remaining abandoned metal mines that exceed environmental standards (KMOE 2017a).

In 2004, media reports of an outbreak of itai-itai disease-like symptoms due to Cd exposure among residents living near an abandoned metal mine in Goseong County in South Gyeongsang Province drew attention to the need for health impact assessments in areas around abandoned metal mines (Kim et al. 2008). Accordingly, in 2007, the KMOE and the National Institute of Environmental Research (NIER) conducted preliminary studies of 358 metal mines with health concerns due to soil contamination and established long-term plans. Subsequently, community health impact assessments were conducted on residents living in areas near 142 abandoned metal mines between 2008 and 2017. Among the results of community health impact assessments completed in 2014, the average geometric mean concentrations of heavy metals exposed to residents in Indae abandoned metal mine area were 1.92 µg/L in cadmium (Cd), 2.09 µg/dL in blood lead (Pb), and 148.9 µg/L in urinary harmful As (NIER 2014). Exposure level of harmful As was very high, and the health effects of exposure to arsenic were concerned. Therefore, in order to reduce the arsenic exposure of residents in this area, we aimed to reassess the concentration harmful As and investigate the cause of urinary arsenic exposure using stable Pb isotope analysis method.

## Materials and methods

### Individuals and study period

**Fig. 1:**
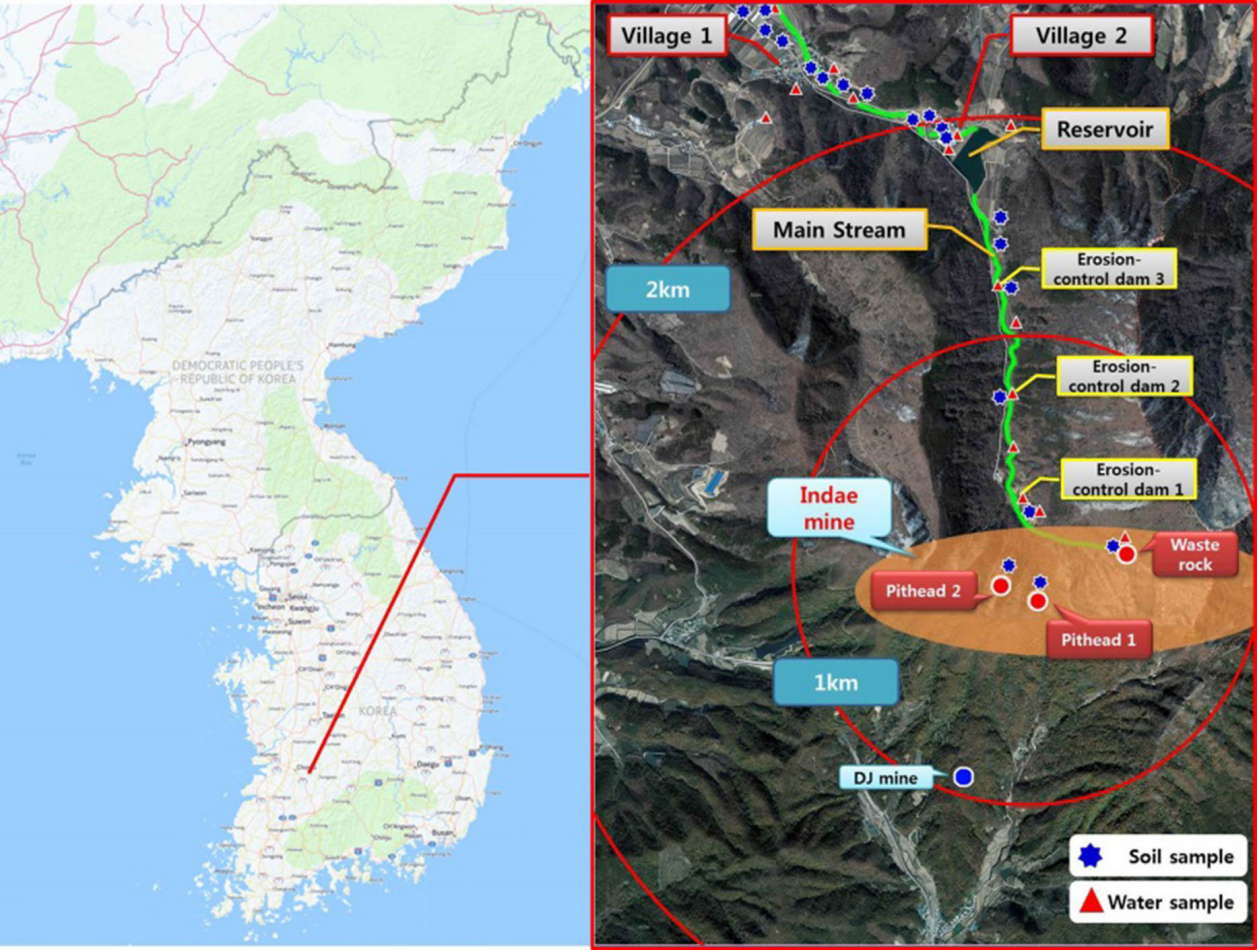
Study area and sampling sites

The present study was performed in the area around the Indae abandoned metal mines, which had shown high urinary concentrations of harmful As among the abandoned metal mines areas surveyed in the “Environmental and health effects survey of residents around 2nd phase abandoned metal mines (II)” conducted in 2014 (Fig. 1) (NIER 2014).

The contents of the present study, including academic and ethical aspects, were approved by the Institutional Review Board (IRB) of the NIER.

**Fig. 2:**
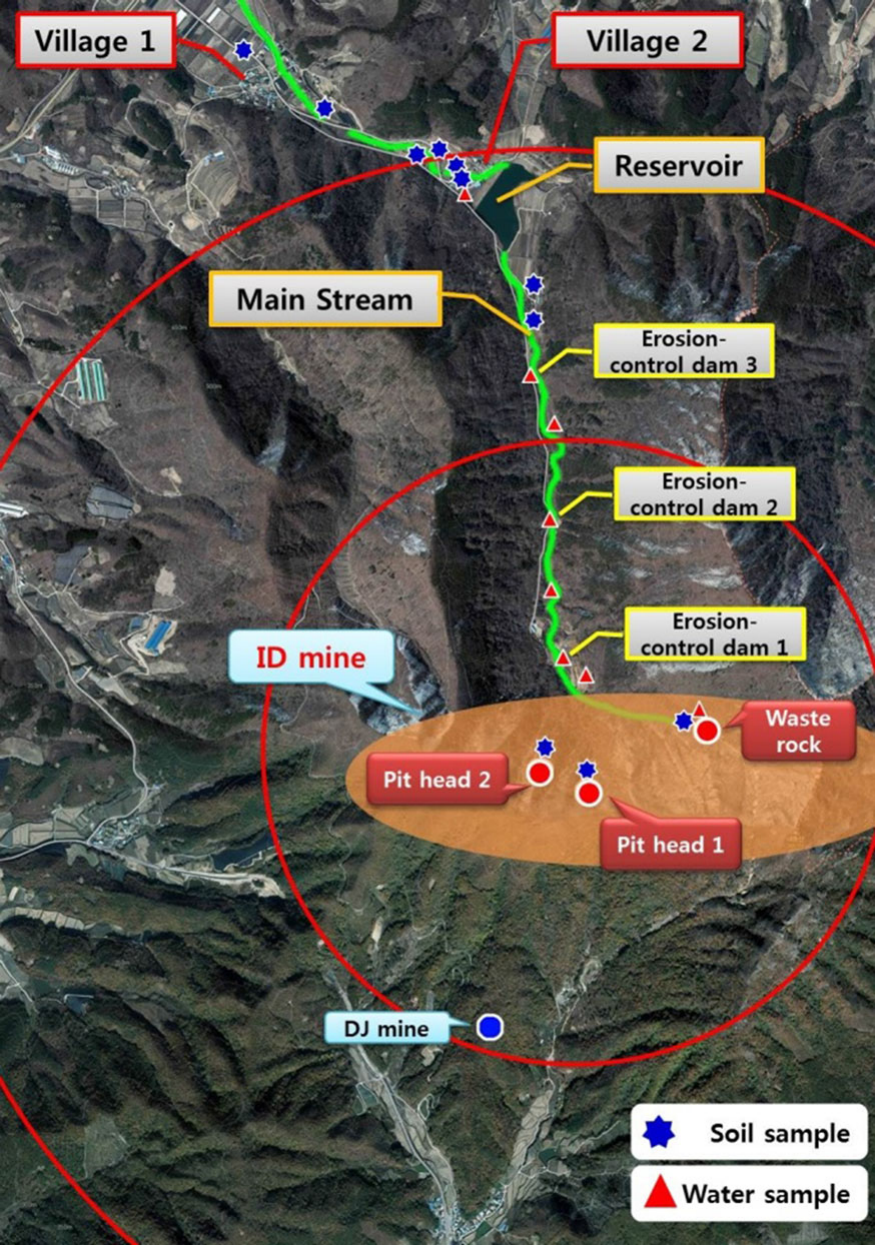
Sites in the Indae abandoned metal mines area exceeding the environmental standards for As

Surveys were conducted in March and May 2015. After explaining the study objective, content, and methods and the right to withdraw their consent to the residents, consent was obtained for their participation in this study and for the use of personal information (Fig. 2).

### Questionnaire survey

To identify As exposure factors, one-on-one interviews were conducted to investigate the demographic and socioeconomic characteristics of participants, in particular their work history in relation to the abandoned metal mine, lifestyles, food self-sufficiency rates, and dietary habits (Table 1).

**Table 1:** Questionnaire survey items

Category	Factors
Personal information	Name, sex, birth data, age
Occupation and exposure history	Past and recent occupation, heavy metal exposure status, residence period in mine area, pesticide use status
Abandoned mine experience	Working experience, working period
Smoking and drinking	Smoking habits, alcohol consumption habits
Disease history	Diagnosis of chronic disease, acute disease and cancer
Dietary habits	Drinking water type, the self-sufficiency rate of agricultural products, seafood intake and frequency, status of seafood intake during the last week

### Sampling and analysis of environmental and urine samples

For identification of As exposure factors and re-assessment of exposure levels in the residents, environmental and urine samples were collected and analyzed. Among the environmental samples, 46 soil samples were collected from areas near the pithead (*n* = 10), from river sediment (*n* = 9), and from farmland the participants were personally farming (*n* = 27). In addition, 31 water samples from areas near the pithead (*n* = 5), from river water (*n* = 22), and from drinking water (*n* = 4) were collected. Additionally, rice grown for self-sufficiency (*n* = 20) was also sampled.

Soil samples were pretreated by Gerhardt block digestion system. Each sample was slowly oxidized by adding 21 mL HCl and 7 mL HNO_3_ while being left at room temperature for 2 h, and then it was decomposed at 180 °C for 2 h, the As content of the soil samples was analyzed by inductively coupled plasma-optical emission spectroscopy (ICP-OES) (Optima 5300 DV, Perkin Elmer) using the Environmental Standard Test Method in Korea (NIER 2015). After the water samples, including river water, were decomposed with nitric acid, the As content was also analyzed by ICP-OES (Optima 5300 DV, PerkinElmer) using the Environmental Standard Test Method in Korea (NIER 2015). After rice samples were pretreated by adding 7 mL HNO_3_ and 1 mL H_2_O_2_ for 30 min in a microwave digestion system, the As content of the rice samples was analyzed by inductively coupled plasma-mass spectrometry (ICP-MS) (Agilent 7500, Agilent Technologies) using the hazardous materials testing method (KMFDS 2015) from the Korean Food Standards Codex.

Urine samples were collected in specimen-cup directly from participants at questionnaire survey time. The samples were transported to the laboratory below 4 °C and stored in an ultra-low temperature freezer at − 70 °C until analysis. For more than 1 h prior to analysis, the samples were mixed using a roll mixer, and urinary As was analyzed by a combination of high-performance liquid chromatography (HPLC) (Flexar, PerkinElmer) and ICP/MS (ELAN DRC-e, PerkinElmer) for As speciation analysis. The chemical species were detected in the order of As(III), As(V), MMA, DMA, AsB, and AsC, with method detection limits of each was 0.4, 0.6, 0.5, 0.3, 0.6, and 0.7 μg/L, respectively.

To prevent polyatomic interference by ^40^Ar^35^Cl^+^ or other factors due to the reaction between argon gas and chlorine in the urine samples during the ICP/MS quantification, ultra-high purity oxygen was introduced into a dynamic reaction gas cell (DRC) to convert As in the samples into AsO^+^(*m*/*z* = 91) for quantification. The concentration of harmful As was derived as the sum of As(V), As(III), MMA, and DMA concentrations.

### Analysis of the stable lead isotope ratio in the soil

To assess the association between the soil in the area around the Indae abandoned metal mines and the soil in the residential area, the distribution patterns of Pb and As concentrations in the soil and a stable Pb isotope ratio were analyzed.

The Pb and As concentration patterns were analyzed using ICP-MS (ELAN DRC-e model, PerkinElmer), after the soil samples were pretreated with an environmental standard test method in Korea (NIER 2015). The distribution patterns were analyzed using the US Environmental Protection Agency (EPA) FALCON technique (Plumb 2004).

The stable Pb isotope ratio was analyzed by MC-ICP-MS (Nu plasma II model, Nu Instruments) with samples that were pretreated as described by Choi et al. (2007) and Yoo et al. (2014), in which the samples were introduced with a cyclonic type nebulizer in a wet plasma mode. For instrumental mass fractionation, thallium (T1), the internal standard, was injected to derive the mass fractionation coefficient of T1 (Eq. 1), which was applied to Pb for calibration (Eq. 2).

1 $$f_{T1} = \frac{{\ln (R_{T1} /r_{T1} )}}{{\ln (M_{205} /M_{203} )}}$$

2 $$R_{Pb} = r_{Pb} \left( {\frac{{M_{1} }}{{M_{2} }}} \right)^{{f_{T1} }}$$

### Statistical data analysis

We used SPSS^®^ ver. 20.0 (IBM Korea) to perform the statistical analysis. The correlations among categorical variables were analyzed using *χ*^2^-test (Chi-squared test), and Fisher’s exact tests were performed for cells with an expected observed value < 5. Chi-squared test is a statistical method based on the Chi-square distribution, used to determine whether is a significant difference between the expected frequencies and the observed frequencies in one or more categories. The mean differences between groups were tested using independent-samples *t*-tests and one-way analysis of variance (ANOVA). Urine As concentrations with lognormal distributions were analyzed after logarithmic transformation.

## Results

### Study area and participants

The Indae abandoned metal mines comprised copper, lead, and zinc mines, with five pitheads at the time of development; however, the mining rights expired in 1992. Significant waste accumulated on the steep slopes near the pithead and the nearby ridgeline presented the possibility of runoff, but the likelihood of waste being introduced into the farmland below was low due to three erosion-control dams located below the mines. However, streams with flowing water near the piles of mine waste created the possibility for contaminants to pass to reservoirs below the mines, indicating a neglected potential source of contamination, as there are no mining hazard prevention measures in place to deal with this issue.

Two villages are located within a 2-km vicinity of the Indae abandoned metal mines, where the residents mostly farm rice and mushrooms. Prior to November 2014 when a multi-regional water supply was implemented, river water was used as the source for drinking, agricultural, and municipal water, while river water remains in use as agricultural water even after the implementation of the multi-regional water supply.

A total of 88 people living in 43 households lived in the villages within the area of influence at the end of 2014, based on national resident registration data. Although the present study aimed for a complete enumeration survey, 50 residents finally participated in the survey, due to inpatients and residents living in other areas.

### Questionnaire survey

The survey was conducted twice. The first survey, in March 2015, involved 50 residents, while the second survey, in May 2015, included 43 of the 50 residents who had participated in the first survey. Among the residents who participated in both the first and second surveys, 27 had also participated in the “Environmental and health effects survey of residents around 2nd phase abandoned metal mines (II)” conducted in 2014.

The mean age of the participants was 66.8 years and the mean residence period was 43.6 years. The percentage of residents who currently worked in agriculture was 74.0% (*n* = 37) and the percentage of those with a previous history of working in the mines was 10.0% (*n* = 5). Among the residents with a previous history of working in the mines, one resident had been a chemical demolition specialist with approximately 24.5 years of work experience, while the other four residents had worked for an average of 0.8 years in the mines (Table 2). In relation to their current occupations, 76.0% (*n* = 38) of the residents had experience handling pesticides, with an average experience time of 26.2 years (Table 2).

**Table 2:** Basic characteristics of the surveyed participants in the area around Indae abandoned metal mines

Factors	Male (*n* = 24)	Female (*n* = 26)	Total (*n* = 50)
Age^a^ (years)	66.13 ± 10.15	67.35 ± 11.82	66.76 ± 10.96
Residence period			
< 40	7 (29.2)	12 (46.2)	19 (38.0)
40–59	4 (16.7)	4 (15.4)	8 (16.0)
≥ 60	13 (54.2)	10 (38.5)	23 (46.0)
AM ± SD^a^ (year)	47.00 ± 25.97	40.54 ± 28.13	43.64 ± 27.04
Occupation			
Sales worker	2 (8.3)	1 (3.8)	3 (6.0)
Farmer	20 (83.3)	17 (65.4)	37 (74.0)
Housewife	–	3 (11.5)	3 (6.0)
Unemployed	2 (8.3)	5 (19.2)	7 (14.0)
Abandoned mine experience			
Working experience (yes)	3 (12.5)	2 (7.7)	5 (10.0)
Working period (years)	8.83 ± 13.57	0.63 ± 0.53	5.55 ± 10.60
Smoking habits			
Current	5 (20.8)	1 (3.8)	6 (12.0)
Past	13 (54.2)	2 (7.7)	15.(30.0)
Never smoked	6 (25.0)	23 (88.5)	29 (58.0)
Alcohol consumption habits			
Current	17 (70.8)	16 (61.5)	33 (66.0)
Past	4 (16.7)	1 (3.8)	5 (10.0)
Never consumed	3 (12.5)	9 (34.6)	12 (24.0)
Pesticide use			
Used (yes)	21 (87.5)	17 (65.4)	38 (76.0)
Average period^a^ (year)	28.59 ± 18.02	23.15 ± 14.67	26.23 ± 16.60

Unit: person (%)

^a^*AM* ± *SD*, arithmetic mean ± standard deviation

A multi-regional water supply was implemented in the area around the Indae abandoned metal mines in November 2014. Prior to the implementation of this water supply, 96% (*n* = 48) of the participants had used a simplified water supply as drinking water for an average of 35.8 years. After its implementation, 72.0% (*n* = 36) of the participants used the multi-regional water supply for drinking water. Residents who lived close to the metal mines did not have access to the multi-regional water supply as of March 2015; as a result, 14 participants still used groundwater and/or a simplified water supply for drinking water (Table 3). However, because water from sources other than those near the mines was used for drinking water, As was not detected in these samples (Table 4).

**Table 3:** Dietary questionnaire results of participants residing around the Indae abandoned metal mine

Factors	Male (*n* = 24)	Female (*n* = 26)	Total (*n* = 50)
Drinking water type (before Nov. 2014)			
Tap/mineral water	1 (4.2)	1 (3.8)	2 (4.0)
Simple water supply	23 (95.8)	25 (96.2)	48 (96.0)
Period^a^ (year)	39.74 ± 24.84	31.32 ± 24.05	35.76 ± 24.49
Current drinking water type			
Tap/mineral water	18 (75.0)	18 (69.2)	36 (72.0)
Simple water supply	6 (25.0)	8 (30.8)	14 (28.0)
Rice			
All self-sufficiency	19 (79.2)	20 (76.9)	39 (78.0)
Some purchase	1 (4.2)	2 (7.7)	3 (6.0)
All purchase	4 (16.7)	4 (15.4)	8 (16.0)
Fish intake frequency			
< 1 time/week	16 (66.7)	20 (76.9)	36 (72.0)
Over 2 times/week	8 (33.3)	6 (23.1)	14 (28.0)
Fish intake			
< 1	16 (66.7)	18 (69.2)	34 (68.0)
More than one	8 (33.3)	8 (30.8)	16 (32.0)
Seaweed intake frequency			
< 1 time/week	19 (79.2)	21 (80.8)	40 (80.0)
Over 2 times/week	5 (20.8)	5 (19.2)	10 (20.0)
Eat seafood during the last week (yes)	19 (79.2)	20 (76.9)	39 (78.0)
Eat seafood during the last 3 days (yes)	14 (58.3)	19 (73.1)	33 (66.0)
Ingested seafood: fish, shellfish, shrimp	8 (57.1)	7 (36.8)	15 (45.5)
Ingested seafood: seaweed	13 (92.9)	18 (94.7)	31 (93.9)
Ingested seafood: fish and seaweed	7 (53.8)	6 (33.3)	13 (41.9)

Unit: person (%)

^a^*AM* ± *SD*, arithmetic mean ± standard deviation

**Table 4:** Arsenic concentrations in environmental samples taken from the area around the Indae abandoned metal mines

Factors	*N*	As concentration		
		AM ± SD^a^	Median	Min–Max
Soil				
Pithead (mg/kg)	10	9144.2 ± 8509.1	10233.0	64.1–25236.1
River sediment (mg/kg)	9	1759.8 ± 2734.1	578.9	96.9–7357.2
Agricultural land (mg/kg)	27	22.4 ± 11.3	21.5	5.7–56.3
Water				
Pithead (mg/L)	5	0.2 ± 0.2	0.3	0.0–0.4
River water (mg/L)	22	0.2 ± 0.4	0.04	0.0–1.8
Drinking Water (μg/L)	4	ND^b^	ND^b^	ND^b^
Rice grain (mg/kg)	20	0.16 ± 0.05	0.15	0.1–0.3

^a^Arithmetic mean ± standard deviation

^b^Not detected

In relation to food consumption, the self-sufficiency rate in terms of rice consumption was 84.0% (*n* = 42). With respect to seafood consumption, most participants consumed approximately one fish or less, less than once a week and, also consumed seaweeds less than once a week. Among the participants, 39 (78.0%) had consumed seafood within 1 week of the survey; among them, 33 had consumed seafood within 3 days of the survey. Among the participants who had consumed seafood within 3 days of the survey, 31 had consumed seaweeds and 13 had consumed both fish and seaweed (Table 3).

As the consumption of seafood can affect the arsenic concentration in urine, although the participants were notified not to consume seafood for 3 days prior to the survey, the consumption of seafood could not be properly controlled for since residents have a lifestyle of gathering at the community center to share meals, except for breakfast.

### Analysis of the environmental samples

To identify the As exposure factors in relation to the residents, 46 soil samples from areas near the pithead, from river sediment, and from farmland belonging to the participants, as well as 31 water samples from areas near the pithead, from river water, from the simplified water supply used as drinking water, and 20 rice samples from rice grown for self-sufficiency, were collected (Table 4).

Among 46 soil and sediment samples, 28 samples exceeded the defined worrisome levels of soil contamination in Korea (KMOE, area 1: 25 mg/kg; area 2: 50 mg/kg) (KMOE 2016), which may be of concern for human health and dwellings and for plant and animal growth. Sixteen of these samples also exceeded the countermeasure levels of soil contamination in Korea (KMOE, area 1: 75 mg/kg; area 2: 150 mg/kg) that required measures against soil pollution that inhibits the growth of animals and plants (KMOE 2016) (Table 5). The agricultural soil samples that exceeded the environmental standards in Korea were areas that had experienced flooding.

**Table 5:** Sediments and soils sites exceeding the environmental standards for As

Sample	Sampling site	Class	As concentration (mg/kg)
Sediment	Waste rock 1	Area 2	**15738.50**
	Waste rock 2	Area 2	**10438.90**
	Waste rock 3	Area 2	**1138.82**
	Waste rock 4	Area 2	*64.09*
	Waste rock 5	Area 2	*65.07*
	Erosion-control dam 1	Area 2	*96.92*
	Erosion-control dam 2	Area 2	**636.42**
	Erosion-control dam 3	Area 2	**5661.06**
	Erosion-control dam 4	Area 2	**480.78**
	Erosion-control dam 5	Area 2	**578.86**
	Floodgate	Area 2	**7357.18**
	Stream 1	Area 2	**673.77**
	Stream 2	Area 2	**202.47**
	Stream 3	Area 2	**151.08**
Soil	Waste rock 6	Area 2	**14097.00**
	Waste rock 7	Area 2	**25236.10**
	Pithead 1	Area 2	**10027.10**
	Pithead 2	Area 2	**738.38**
	Pithead 3	Area 2	**13897.80**
	Field 1	Area 1	*36.89*
	Paddy 1	Area 1	*42.33*
	Field 2	Area 1	*32.79*
	Field 3	Area 1	*56.33*
	Paddy 2	Area 1	*27.47*
	Field 4	Area 1	*30.89*
	Paddy 3	Area 1	*29.89*
	Paddy 4	Area 1	*26.18*
	Field 5	Area 1	*24.95*
Worrisome levels of soil contamination		Area 1	25
		Area 2	50
Countermeasure levels of soil contamination		Area 1	75
		Area 2	150

Bold and italics indicate samples that exceeded the countermeasure and worrisome levels of soil contamination in Korea, respectively

^a^Area 1: Dry paddy, paddy, orchard, and ranch; mineral spring land; open field (residential); school use and parkland; historic sites; burial grounds; and playgrounds

^b^Area 2: Forest/salt farm/open field (non-residential); mixed-use land including warehouses, rivers, public land, sports-use, recreation-use, and religious-use

Among the 31 water samples, 11 samples from the vicinity of the pitheads and from river water exceeded the water quality environmental standards for As (0.05 mg/L) (KMOE 2018). In particular, water samples from river upstream 3 had been used as drinking water prior to the implementation of the multi-regional water supply had a concentration of 0.161 mg/L, exceeding the water quality standards for drinking water (0.01 mg/L) (KMOE 2017b) by 16-fold (Table 6).

**Table 6:** Sites exceeding the water quality standard of As

Sampling site	Standard^a^ (mg/L)	As (mg/L)	Remarks
Waste rock 1	0.05	0.392	650 m above sea level
Waste rock 2	0.05	0.342	650 m above sea level
Waste rock 3	0.05	0.260	600 m above sea level
Waste rock 4	0.05	0.199	550 m above sea level
River upstream 1	0.05	0.232	Top of erosion-control dam 1
River upstream 2	0.05	0.339	Top of erosion-control dam 1
River upstream 3	0.05	0.161	Drinking water for village 1 in the dry season
Erosion-control dam 1	0.05	0.258	
Erosion-control dam 2	0.05	0.138	
Erosion-control dam 3	0.05	0.095	
Reservoir floodgate	0.05	1.790	

^a^Environmental standards for water quality in the Republic of Korea (KMOE 2017b)

Among the 20 rice samples obtained from rice grown for self-sufficiency, the arithmetic mean concentration of As was 0.16 ± 0.05 mg/kg, which was below the 0.2 mg/kg standard for As in rice set by the Korean Ministry of Food and Drug Safety (KMFDS 2015); however, four samples exceeded the standard. This was slightly higher than the concentration in rice reported by Kim et al. (0.103 ± 0.024 mg/kg) (Kim et al. 2009) (Table 4).

### Analysis of the stable lead isotope ratio

A stable Pb isotope ratio was used to assess the environmental impact of the Indae abandoned metal mine as As does not have isotopes.

**Fig. 3:**
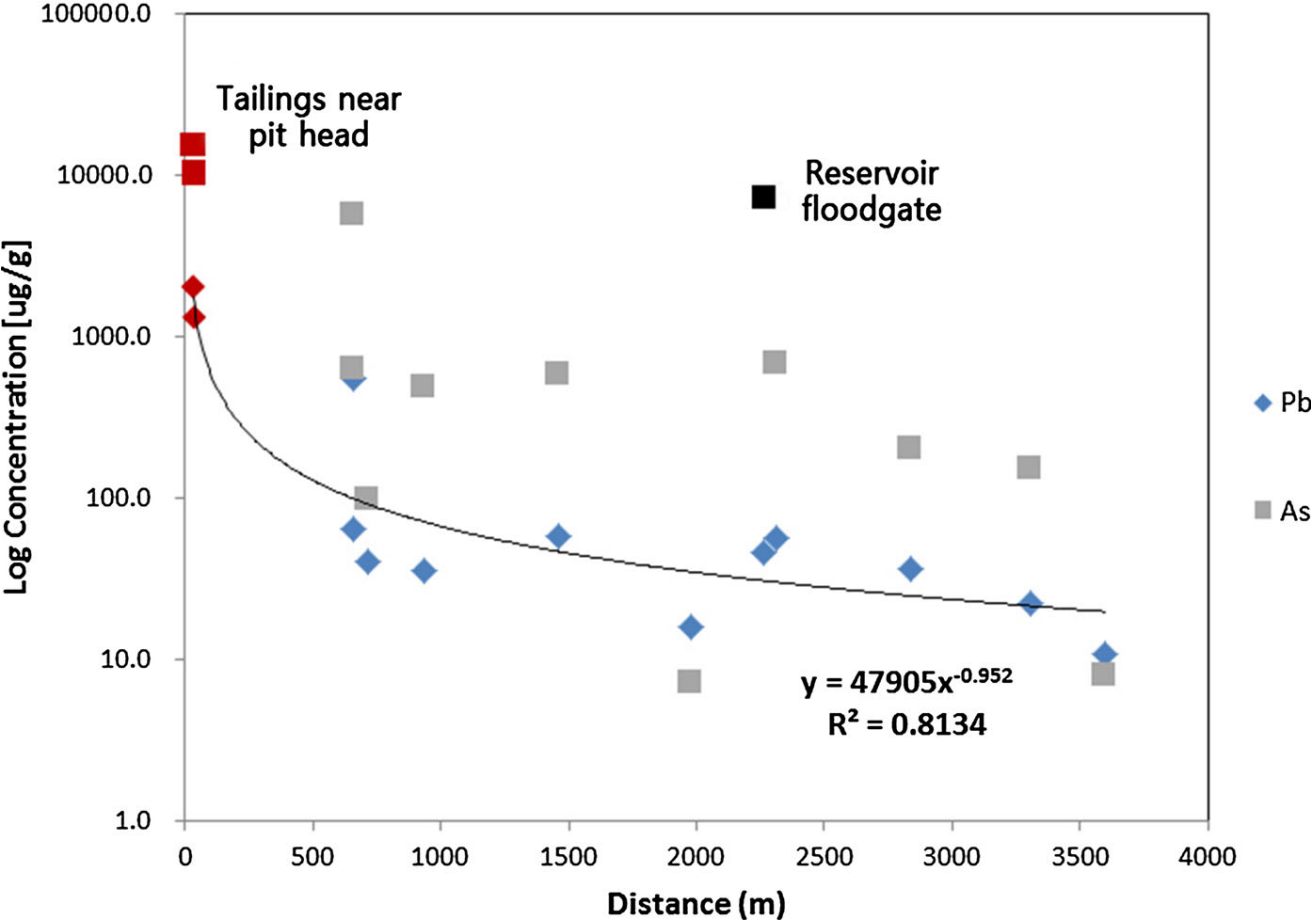
Pb and As concentrations in the soil in relation to the distance from the Indae abandoned metal mines

**Fig. 4:**
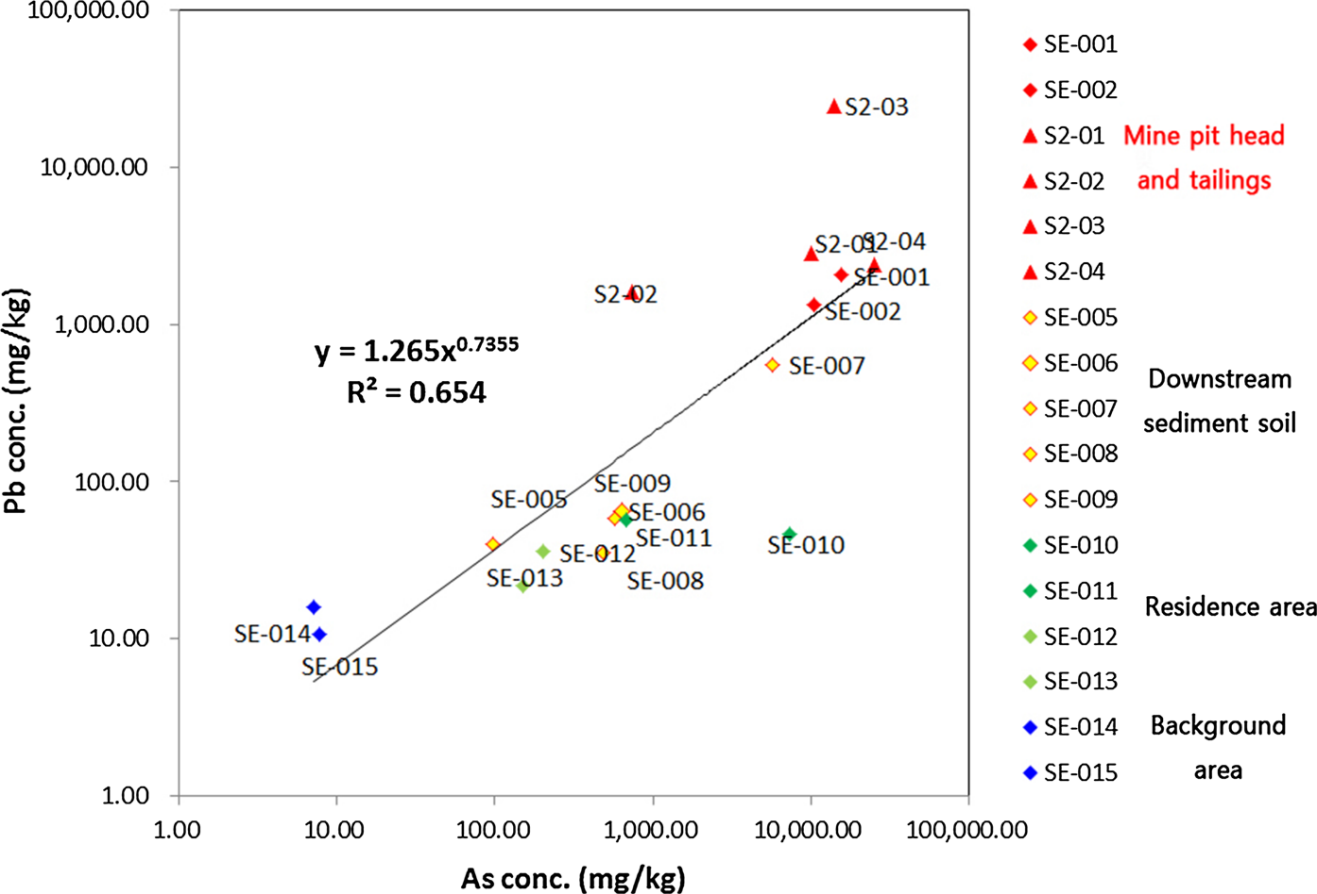
Correlations of Pb and As concentrations in soil samples

An investigation of the correlation between As and Pb concentrations in soil downstream from the Indae abandoned metal mines found that Pb and As concentrations in soil by distance showed a gradually decreasing trend in the downstream direction from the pithead (*R*^2^ = 0.8134) (Fig. 3). Moreover, the correlation between Pb and As concentrations in the soil from the residential areas and the soil downstream from the Indae abandoned mines showed an *R*^2^ of 0.654; since Pb and As behave similarly, the use of the stable Pb isotope ratio for assessment of the source tracking was validated (Fig. 4).

**Fig. 5:**
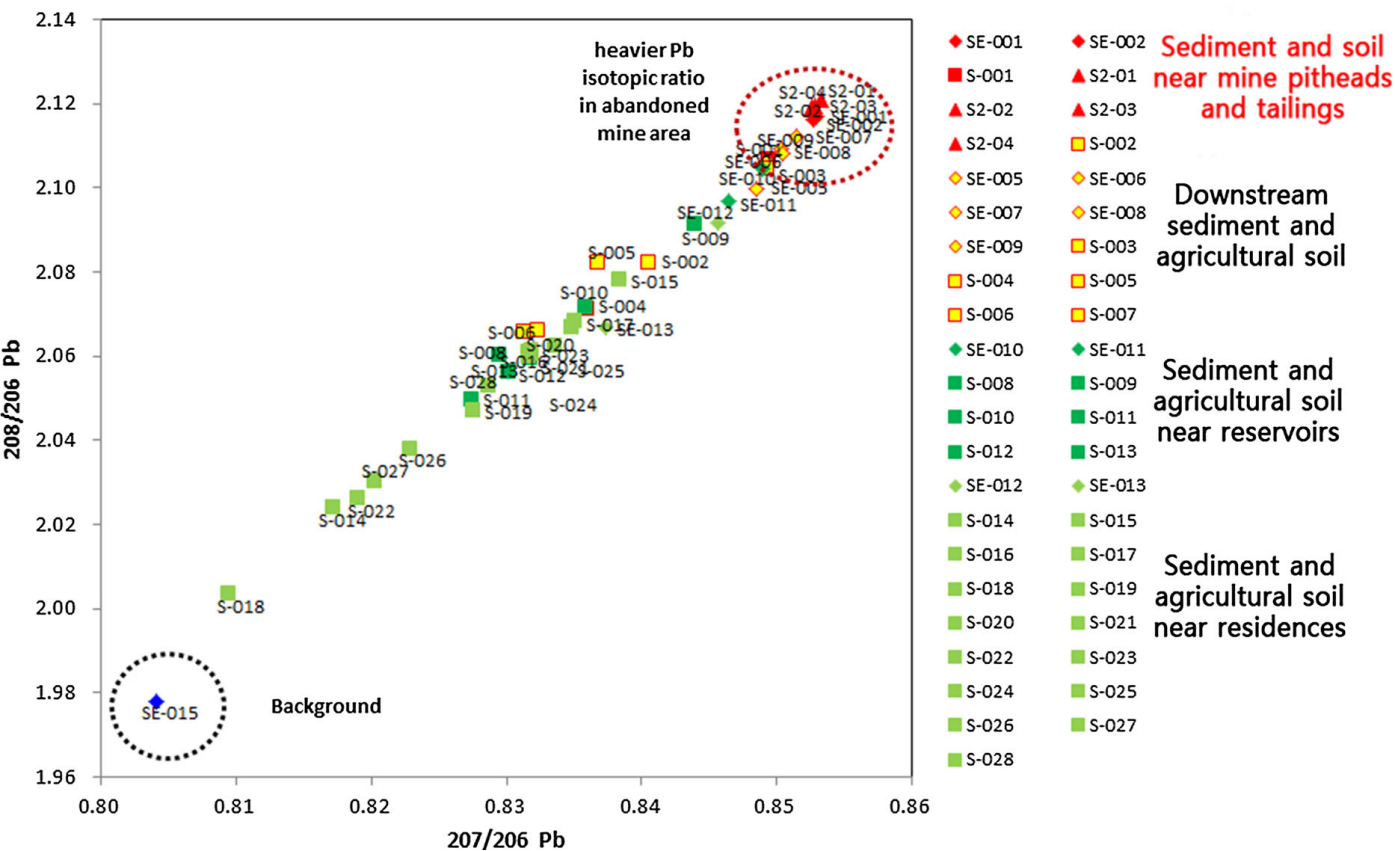
The 3-isotope plot (^207/206^Pb vs. ^208/206^Pb) of soil samples in the Indae abandoned metal mine area

In the 3-isotope plot (^207/206^Pb vs. ^208/206^Pb) of samples from the abandoned metal mines and from mine tailings downstream from the pithead by distance, a stable Pb isotope ratio was located on the same trend line, which confirmed that the soil in the residential area was within the area of influence of the Indae abandoned metal mines (Fig. 5).

### Urinary concentrations of harmful As

The urinary concentrations of harmful As in 39 residents of the Indae abandoned metal mines area are shown in Table 7. Although 50 residents participated in the first survey, a urine sample could not be collected from one resident and 10 residents showed urinary concentration of creatinine that deviated from the WHO reference value (0.3–3.0 g/L) (WHO 1996) Consequently, data from these 11 participants were excluded from the statistical analysis in consideration of the reliability of the results.

**Table 7:** Comparative urinary As concentrations in participants from the Indae abandoned metal mines area

Sex	Indae mines^a^	Abandoned mines		Korea^b^		U.S RV95^c^	Reference values
		2013	2014	Total	≥ 65		ACGIH^d^
Total	31.9	48.6	39.1	35.0	40.3	52.5	35.0
Male	30.8	48.3	41.3	36.7			
Female	33.4	49.0	38.3	33.4			

Unit: μg/L

^a^As(III) + As(V) + MMA + DMA (Geometric Mean)

^b^1st Korea National Environmental Health Survey (KNEHS) (2009–2011) (NIER 2012)

^c^National Health and Nutrition Examination Survey (NHANES), (Centers for Disease Control and Prevention (CDCs), USA, (CDC 2015). “4th National Report on Human Exposure to Environmental Chemicals”)

^d^American Conference of Governmental Industrial Hygienists (ACGIH 2006). Biological Exposure Indices

The geometric mean urinary concentrations of harmful As was 31.92 μg/L, which was lower than the 148.9 μg/L concentration found in the 2014 survey (NIER 2014), and lower than the average level among Korean adults aged 20 years or older (35.0 μg/L) (NIER 2012) and the results from a survey of abandoned metal mine areas (39.1–48.6 μg/L) (NIER 2013, 2014) (Table 7).

The geometric mean for the chemical species of As were 1.45, 0.74, 2.43, 27.63, and 88.62 μg/L for As(III), As(V), MMA, DMA, and AsB, respectively. Among these, the concentration of inorganic As, (As(III) and AS(V)), was 1.61 μg/L, while the concentration of harmful As combining inorganic As and its metabolites (MMA and DMA) was 31.92 μg/L, of which MMA and DMA accounted for 7.6% and 86.6%, respectively, of total harmful As (Table 8).

**Table 8:** Specific As urinary concentrations in participants from the Indae abandoned metal mines area

Factors	AM ± SD^a^	GM (95% CI)^b^	Min	Max
As(III)	1.66 ± 0.94	1.45 (1.24, 1.71)	0.54	4.19
As(V)	0.75 ± 0.13	0.74 (0.68, 0.81)	0.61	1.05
As(III) + As(V)	1.89 ± 1.18	1.61 (1.35, 1.93)	0.54	4.81
MMA	2.83 ± 1.64	2.43 (2.00, 2.92)	0.87	6.97
DMA	32.31 ± 21.36	27.63 (23.25, 32.53)	8.24	127.12
MMA + DMA	34.99 ± 22.17	30.13 (25.28, 36.07)	9.11	129.64
Arsenobetaine	106.44 ± 75.69	88.62 (73.55, 107.15)	26.93	422.51
Harmful As^c^	36.88 ± 22.81	31.92 (27.67, 37.82)	10.14	132.20

Unit: µg/L

^a^AM ± SD: arithmetic mean ± standard deviation

^b^GM: geometric mean; 95% CI: 95% confidence interval

^c^Harmful As: As(III) + As(V) + MMA + DMA

**Fig. 6:**
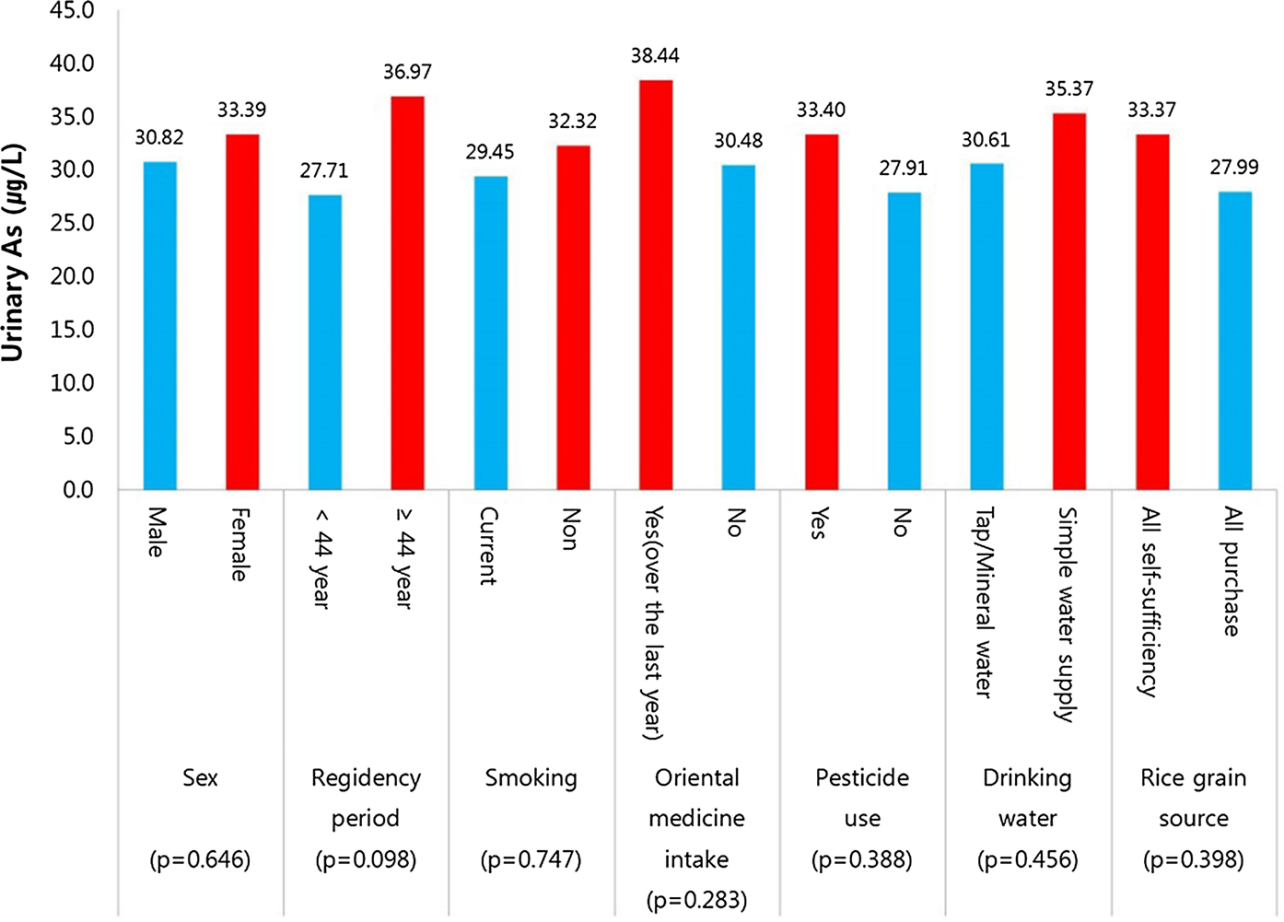
Urinary harmful As concentrations in relation to the individual characteristics of participants from the Indae abandoned metal mines area

Comparison of urinary concentrations of harmful As by major exposure factors from the questionnaire survey revealed higher urinary concentrations of harmful As among residents with a longer residence duration and who consumed rice grown for self-sufficiency; however, the differences were not statistically significant (Fig. 6).

**Fig. 7:**
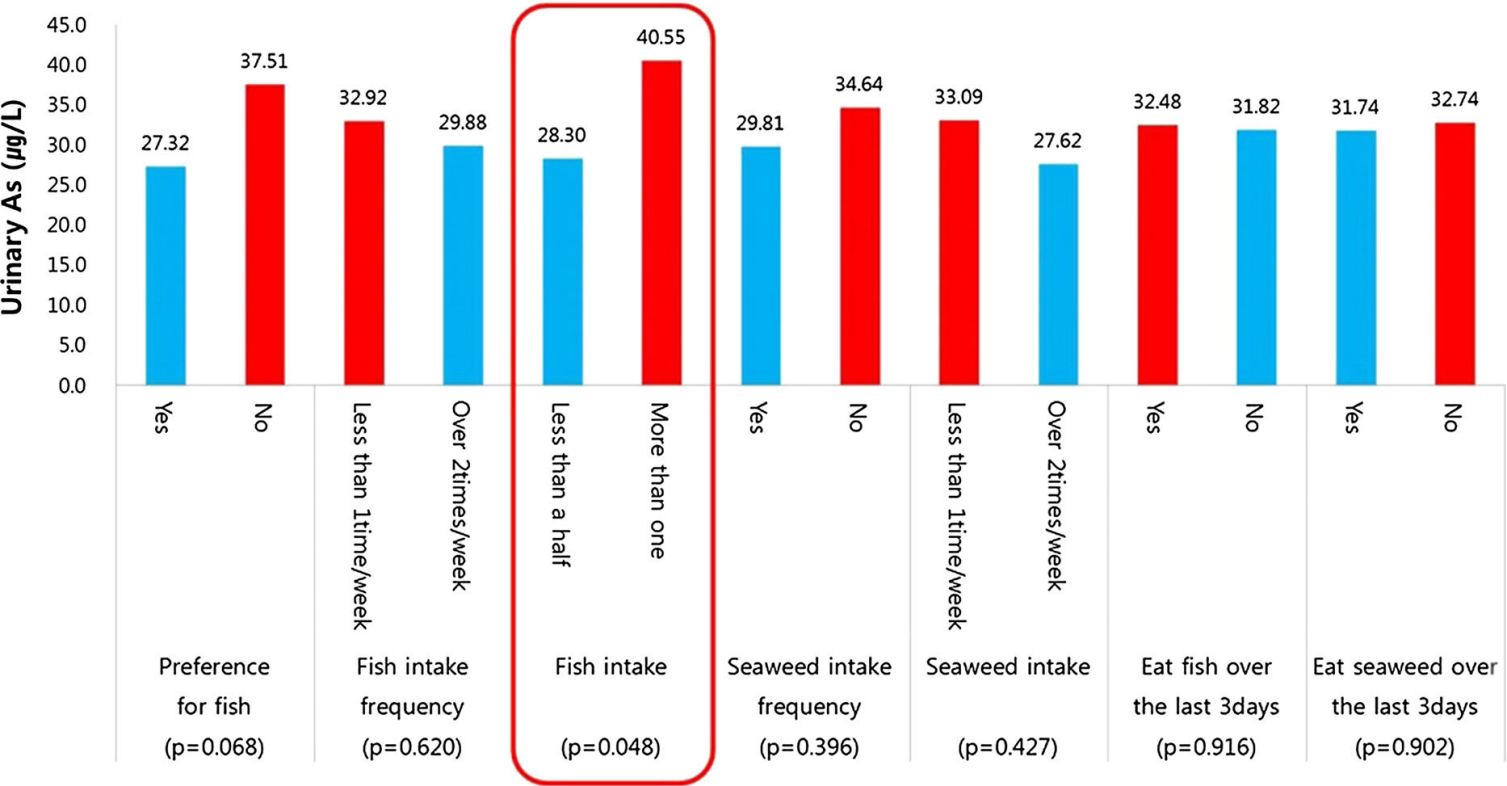
Urinary harmful As concentrations in relation to the dietary characteristics of participants from the Indae abandoned metal mines area

With respect to dietary characteristics, residents who consumed more than one whole fish per sitting had a significantly higher urinary concentration of harmful As than those who consumed less than half a fish per sitting (*p* = 0.048) (Fig. 7).

Urinary concentrations of harmful As in residents of the Indae abandoned metal mines area were surveyed three times, when considering the original 2014 Environmental and Health Effects Survey of Residents around 2nd Phase Abandoned Metal Mines (II) (NIER 2014). A total of 27 residents participated in all three surveys. Among them, 13 were excluded from the analysis for having urinary creatinine concentrations that deviated from the WHO reference value. Meanwhile, 14 participants with normal creatinine concentrations showed significantly higher urinary concentration of harmful As in the original 2014 survey (167.06 µg/L) (NIER 2014) than in the 2015 surveys (1st survey, 32.78 µg/L; 2nd survey, 30.80 µg/L) (Table 9).

**Table 9:** Urinary harmful As concentrations by survey time for the same participants from the Indae abandoned metal mines area

Factors^1)^	2014	2015 1st survey	2015 2nd survey	*p* value^3)^
	(*n* = 14)	(*n* = 14)	(*n* = 14)	
As(III) + As(V)	23.13^a,b^ (13.07, 38.54)	1.70^a^ (1.25, 2.30)	2.86^b^ (2.34, 3.41)	0.000
MMA + DMA	142.55^c,d^ (85.43, 219.51)	30.82^c^ (22.71, 41.46)	28.46^d^ (22.70, 35.57)	0.000
Arsenobetaine	48.07 (33.68, 68.53)	77.77 (59.41, 100.57)	42.79 (28.63, 62.15)	0.000
Harmful As^2)^	167.06^e,f^ (99.67, 263.09)	32.78^e^ (24.35, 43.35)	30.80^f^ (23.89, 38.12)	0.000

Unit: µg/L

^1)^GM: geometric mean; 95% CI: 95% confidence interval

^2)^Harmful As: As(III) + As(V) + MMA + DMA

^3)^One-way analysis of variance (ANOVA)

^a,b,c,d,e,f^Tukey’s Post Hoc Multiple Comparisons, the same letter indicates statistical significance

## Discussion

Mine hazards have been identified in 1217 of 2084 (approximately 56%) abandoned metal mines throughout Korea, the types of mine hazard include soil contamination due to mine tailings and mine waste (MIRECO 2017a, b). In these 1217 mines, mining hazard prevention work has been undertaken on 398 mines since 2007; the remaining mines have yet to be dealt with and with no appropriate prevention measures have been taken to address sources of contamination such as mine waste and tailings waste (MIRECO 2017a, b). The Indae abandoned metal mine area consists of a single water system, with the mine located in a high-altitude area and residential properties located in a low-altitude area within a 2-km radius of the mines. Although some mining hazard prevention work, such as the installation of erosion-control dams and tree planting, has been implemented in this area, the sources of contamination, such as mine waste and tailings near the pitheads remain unaddressed. As in mine waste and tailings can be exposed to the environment (Kim et al. 2005; Yang et al. 2015). Consequently, some soil and water in the low-altitude areas exceeded the environmental standards for As.

The residents of the Indae abandoned metal mine area who participated in the surveys had a mean age of 66.8 years and a mean duration of residence of 43.6 years. Therefore, there is a high probability of long-term exposure to contaminants that have not been removed or contained after the mine was abandoned and, the use of river water contaminated by the abandoned metal mine as drinking water during dry seasons, may affect their health.

Analysis of the soil, sediment, and river water to assess the association between the soil of the Indae abandoned metal mine area and the soil in residential areas confirmed a correlation between Pb and As concentrations in the soil. Since Pb and As behave similarly, the use of the stable Pb isotope ratio for assessment of the pollution source tracking in this area was validated (Figs. 3, 4). In the 3-isotope plot (^207/206^Pb vs. ^208/206^Pb) of soil samples in Indae abandoned metal mine area, a stable Pb isotope ratio was located on the same trend line, which confirmed that the soil in the residential area was within the area of influence of the Indae abandoned metal mines (Fig. 5). Therefore, we judged that the pollution source of As in this area was the Indae abandoned metal mine.

It can be used as a pollution source tracking method in other regions using the analysis of Pb and As concentrations, environmental behaviors and the analysis of stable Pb isotope ratio applied to this study (Choi et al. 2007; Yoo et al. 2014; Ahn et al. 2017).

The urinary concentrations of harmful As in the residents in the 2014 survey, the first survey in 2015, and the second survey in 2015 were 148.9, 31.92, and 30.54 μg/L, respectively, showing a large decrease since the 2014 survey. The most likely explanation for this decrease is that the As exposure risk through drinking water was mitigated following the implementation of a multi-regional water supply in November 2014.

In San Pedro de Atacama, Chile, where the As concentration in river and tap water used as drinking water is 0.13–0.67 mg/L, the mean urinary concentration of harmful As in residents was 582.4 μg/L (638.1 μg/g-Cr), while in Toconao, Chile, where the As concentration in tap water used as drinking water is 0.015 mg/L, the mean urinary concentration was 58.5 µg/L (67.1 µg/g-Cr) (Biggs et al. 1997). With respect to the chemical species of As, the concentrations of inorganic As, MMA, and DMA were 107.6 (18.5%), 90.1 (15.5%), and 384.7 µg/L (66.1%), respectively, in the San Pedro area, and 8.7 (14.6%), 6.1 (10.4%), and 44.1 µg/L (75.0%), respectively, in the Toconao area. In the Health Effects of Arsenic Longitudinal Study (HEALS, 2000–2011) (Wu et al. 2014) from Bangladesh, which examined 1078 adults aged between 27 and 52 years, the concentration of As in well water used as drinking water was 0.076 mg/L and the urinary concentration of harmful As was 258.7 µg/g-Cr (Wu et al. 2014). As these findings indicate, the urinary concentrations of harmful As relative to As concentrations in drinking water tend to vary, which may be attributable to individual and regional differences in the amount of drinking water consumed and individual differences in the ability to metabolize As (Wu et al. 2014).

The significance of the present study is that it identified specific As exposure factors in residents of an area comprising abandoned metal mine where the risks of As exposure can be generally high, and also assessed the association between the soil of abandoned metal mine area and the soil in residential areas. However, the limitations of the present study include the low number of residents in the study area and the low number of participants. However, the results indicated that the exposure to As among the residents of the Indae abandoned metal mine area was due to the exposure of the soil in residential areas and the consumption of river water during the dry season affected by the abandoned metal mine.

## Conclusions

The present study identified As exposure factors among residents of the Indae abandoned metal mines area and assessed the association of those factors with the abandoned metal mines. The urinary concentrations of harmful As in the residents of this area included in this study decreased following the introduction of once a multi-regional water supply, due to a reduced As exposure risk from contaminated drinking water. Moreover, an analysis of the correlation between the soil of residential areas and the soil of the Indae abandoned metal mines area, using a stable Pb isotope ratio confirmed that soil in the residential areas was affected by the proximity to the abandoned metal mines. Since dealing with mine waste should be a priority given the potential health risks, and with As concentrations in agricultural soil and river water still exceeding recognized environmental standards in the study area, environmental management, including the removal of mine waste and the restoration of agricultural soil, is necessary.
